# Production of Tissue-Engineered Skin Substitutes for Clinical Applications: Elimination of Serum

**DOI:** 10.3390/ijms241612537

**Published:** 2023-08-08

**Authors:** Emilie J. Doucet, Sergio Cortez Ghio, Martin A. Barbier, Étienne Savard, Brice Magne, Meryem Safoine, Danielle Larouche, Julie Fradette, Lucie Germain

**Affiliations:** 1 The Tissue Engineering Laboratory (LOEX), Université Laval’s Research Center, Québec, QC G1V 0A6, Canada; emilie.doucet.4@ulaval.ca (E.J.D.); sergio.cortez-ghio.1@ulaval.ca (S.C.G.); martin.barbier.1@ulaval.ca (M.A.B.); etienne.savard.4@ulaval.ca (É.S.); brice.magne@crchudequebec.ulaval.ca (B.M.); meryem.safoine.1@ulaval.ca (M.S.); danielle.larouche@crchudequebec.ulaval.ca (D.L.); julie.fradette@fmed.ulaval.ca (J.F.); 2 Department of Surgery, Faculty of Medicine, Université Laval, Québec, QC G1V 0A6, Canada; 3 Regenerative Medicine Division, CHU de Québec-Université Laval Research Centre, Québec, QC G1V 0A6, Canada

**Keywords:** cell culture, tissue engineering, defined medium, stem cells, skin

## Abstract

Tissue-engineered skin substitutes (TESs) are used as a treatment for severe burn injuries. Their production requires culturing both keratinocytes and fibroblasts. The methods to grow these cells have evolved over the years, but bovine serum is still commonly used in the culture medium. Because of the drawbacks associated with the use of serum, it would be advantageous to use serum-free media for the production of TESs. In a previous study, we developed a serum-free medium (Surge SFM) for the culture of keratinocytes. Herein, we tested the use of this medium, together with a commercially available serum-free medium for fibroblasts (Prime XV), to produce serum-free TESs. Our results show that serum-free TESs are macroscopically and histologically similar to skin substitutes produced with conventional serum-containing media. TESs produced with either culture media expressed keratin 14, Ki-67, transglutaminase 1, filaggrin, type I and IV collagen, and fibronectin comparably. Mechanical properties, such as contraction and tensile strength, were comparable between TESs cultured with and without serum. Serum-free TESs were also successfully grafted onto athymic mice for a six-month period. In conclusion, Surge SFM and Prime XV serum-free media could be used to produce high quality clinical-grade skin substitutes.

## 1. Introduction

Methods to treat severe burn injuries have evolved over time. It is now possible to produce tissue-engineered skin substitutes (TESs), using both fibroblasts and keratinocytes expanded from a small biopsy, providing treatment for patients with extensive burns [1,2,3]. Methods for the in vitro culture and massive expansion of primary keratinocytes were first developed in the 1970′s by James Rheinwald and Howard Green [4,5]. These procedures rely on the use of a feeder layer combined with a medium containing bovine serum. Although there have been several changes in the culture methods over the years, bovine serum remains widely used for the production of TESs [2,3,6]. Serum is the part of the blood that does not contain cells, platelets, and clotting factors [7]. Serum is the most common supplement in cell culture media due to its numerous functions [8]. It contains essential components, such as hormones, vitamins, growth factors, and transport and attachment proteins [7,9,10,11]. It is also thought to be a key component in the preservation of epithelial stem cells, which is crucial to ensure long-term skin substitute persistence after grafting [6].

Serum has been utilized for decades. However, the use of serum has drawbacks from both a scientific and biosafety point of view. Despite efforts to identify all of its components, serum has never been fully characterized [12,13]. It is a complex mixture, and the concentration of several components may vary drastically from one batch to another. This variability can lead to differences in cell growth and cell product consistency that may interfere with the reproducibility of experiments [7,8,14]. There is also a risk of introducing contaminants into the final product because serum can possibly be contaminated with mycoplasmas, prions, and viruses [9,15]. A study showed that burn patients treated with keratinocyte grafts expressed a significant antibody response to serum proteins [16]. Therefore, it is necessary to test every batch before use and submit it to rigorous quality controls.

In a previous study, our team developed a serum-free medium (Surge SFM) suitable for the isolation and culture of primary keratinocytes and the production of TESs. The TESs produced using Surge SFM were grafted onto a mouse model of an excisional wound and were shown to persist and regenerate the epidermis in a six-month follow-up study. However, in these experiments, the tissue-engineered dermis was produced by culturing fibroblasts in a medium containing serum [17]. A tissue-engineered dermis was produced by the self-assembly approach in the absence of serum [18], but the addition of an epidermis to produce TESs with serum-free media has not been achieved yet.

The objective of this study was to produce TESs with both dermal and epidermal layers cultured entirely in serum-free media and to evaluate the quality of these substitutes both in vitro and in vivo. The long-term aim is to translate these findings to the production of TESs for clinical applications.

## 2. Results and Discussion

### 2.1. Fibroblasts and Epithelial Cell Culture in Serum-Free Media

**Figure 1:**
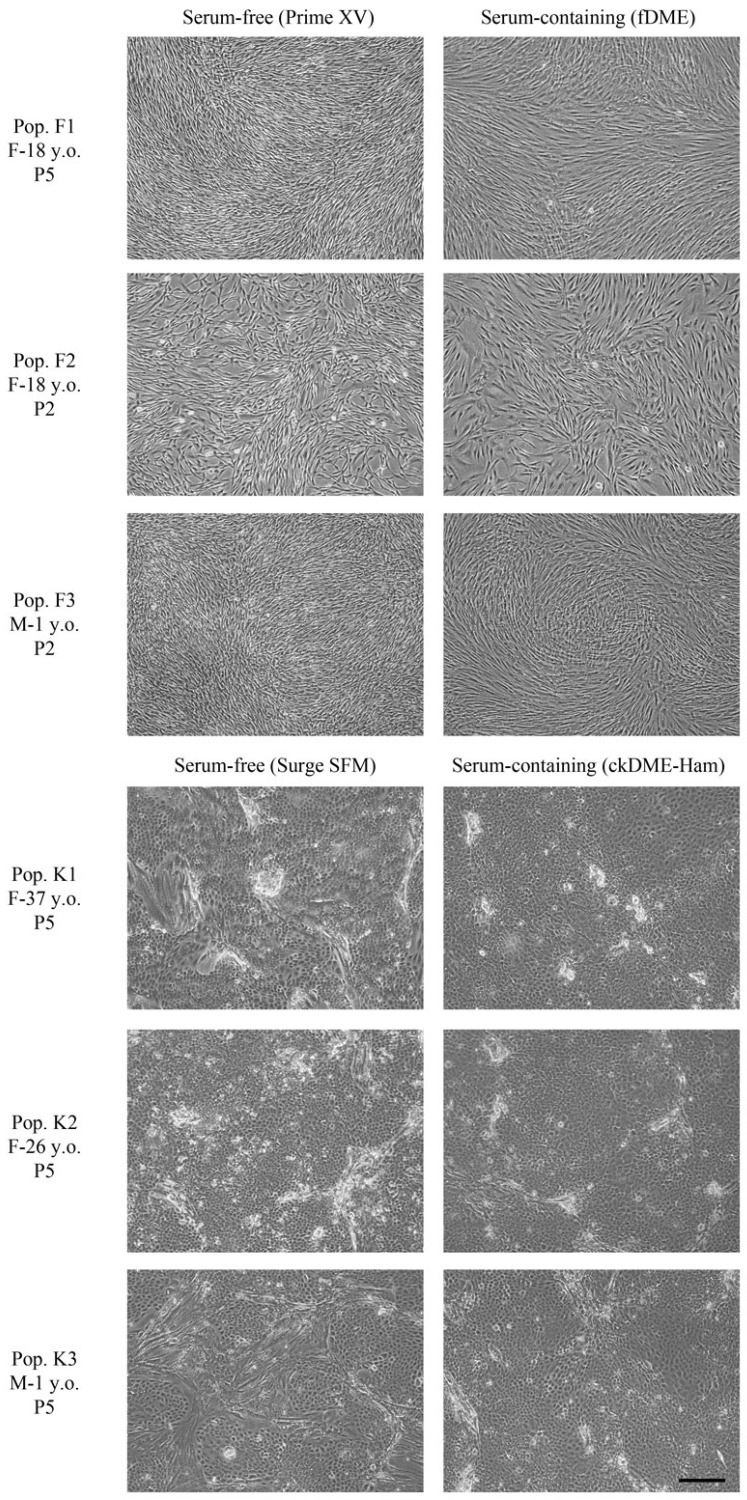
Phase contrast microscopy of fibroblasts (F) and keratinocytes (K) from different donors cultured in serum-free or serum-containing medium. Fibroblasts from population 1 are at passage 5, and population 2 and 3 are at passage 2. The three populations of keratinocytes are at passage 5. F: female; M: male; y.o.: year(s) old; P: passage. Scale bar: 200 μm.

We first wanted to verify if serum-free media could be used to thaw and culture cryopreserved cells as effectively as their serum-containing counterparts. We therefore cultured fibroblasts with Prime XV, a commercially available serum-free medium for fibroblasts, and compared it to fDME serum containing conventional medium. In parallel, we compared Surge SFM serum-free medium to ckDME-Ham, a serum-containing medium for keratinocyte culture. Fibroblasts and keratinocytes expanded in serum-free media displayed a morphology similar to those cultured in serum-containing media (Figure 1). Keratinocytes cultured in Surge SFM and ckDME-Ham demonstrated a similar growth rate, reaching a confluence between 90% and 95% after 5 days and a mean cell size of 16.2 μm (standard deviation (SD) = 0.28) and 16.5 μm (SD = 0.21), respectively. The mean size of fibroblasts cultured in Prime XV and fDME after one passage was 14.98 μm (SD = 0.98) and 18.18 μm (SD = 1.36), respectively. Fibroblasts cultured in Prime XV were smaller and grew faster than those cultured in fDME. This is consistent with results for fibroblasts and other mesenchymal cells previously published by others and us [18,19,20]. The enhanced cell growth in serum-free medium could allow a reduction in culture time and thus be advantageous in a clinical setting. Since cell number is often a limiting factor for clinical applications, the smaller cell size could also be of interest, as it will result in a greater number of cells per flask after cell expansion [18]. This is also what we observed with a number of 13.8 M (SD = 4.6) cells per 75 cm^2^ flask for fibroblasts cultured in serum-containing medium, compared to 29.7 M (SD = 3.6) cells for fibroblasts cultured in serum-free medium. For clinical applications, it is essential to make sure that the cells used to produce a tissue-engineered graft are of great quality and display a normal morphology and expansion rate. Our results suggest that Prime XV and Surge SFM meet these needs and are thus promising media for the culture of fibroblasts and keratinocytes in serum-free conditions.

### 2.2. General Aspects of the Skin Substitutes

**Figure 2:**
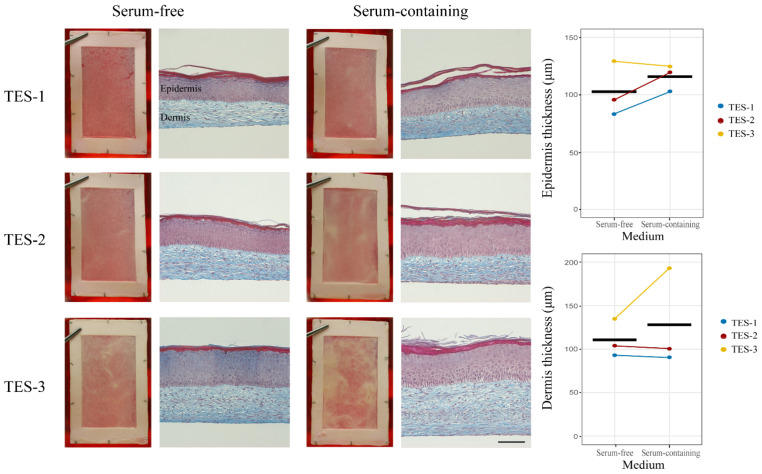
Macroscopic and histological analysis of TESs produced in vitro and cultured 10 days at the air–liquid interface. Representative macroscopic and Masson’s trichrome histological pictures of TES-1, TES-2, and TES-3 produced with keratinocytes and fibroblasts from different donors (see Table 1) using serum-free or serum-containing media. The solid black line represents the mean. Scale bar for histological pictures: 100 μm. Epidermal and dermal thickness was measured using microscopic images taken with Zeiss Axio Imager M2 microscope with an AxioCam ICc1 camera and analyzed with the AxioVision software v4.8.2.0 (Zeiss Canada Ltd., Toronto, ON, Canada). Linear mixed-effect model with paired samples was used to compare the effect of media on thickness. No statistically significant effect of condition was detected (*p* > 0.05).

Next, we investigated the possibility of producing TESs using serum-free media. For this, we thawed and cultured fibroblasts from two donors in Prime XV medium and keratinocytes from three different donors in Surge SFM medium (Table 1) and produced TESs using the self-assembly method [21]. As a control for each donor, we also produced TESs with the serum-containing media that are used in ongoing clinical trials (https://clinicaltrials.gov/study/NCT02350205, 26 January 2015 [2]). After 10 days of in vitro maturation at the air–liquid interface, all TESs (TES-1, TES-2, and TES-3, see Table 1) produced with keratinocytes and fibroblasts from different donors cultured with either serum-free or serum-containing media displayed the expected macroscopic appearance of self-assembled bilayered skin substitutes. However, the intensity and uniformity of the whitish color that develops over time during culture at the air–liquid interface varied between cell donors. This color corresponds to the presence of the stratum corneum, indicating that the epidermal cells have terminally differentiated (stratum corneum). In some cases, certain areas appeared denser or brighter (Figure 2).

Histologically, TESs cultured with either serum-containing or serum-free media exhibited an epidermis covering the dermal component. All typical layers of the human epidermis were present. The stratum basale was organized in the typical cuboidal cell layer in palisade fashion. The stratum spinosum, stratum granulosum, and stratum corneum were also present in all conditions (Figure 2). The thickness of the epidermis and dermis was not significantly different between the two conditions (*p*-value of 0.2814 and 0.4830, respectively). The dermal thickness and the epidermal thickness of TES-3 were greater than those of the two others (Figure 2). That is expected, since cells from a newborn, such as TES-3, are more proliferative and produce thicker substitutes compared to adult cells.

**Figure 3:**
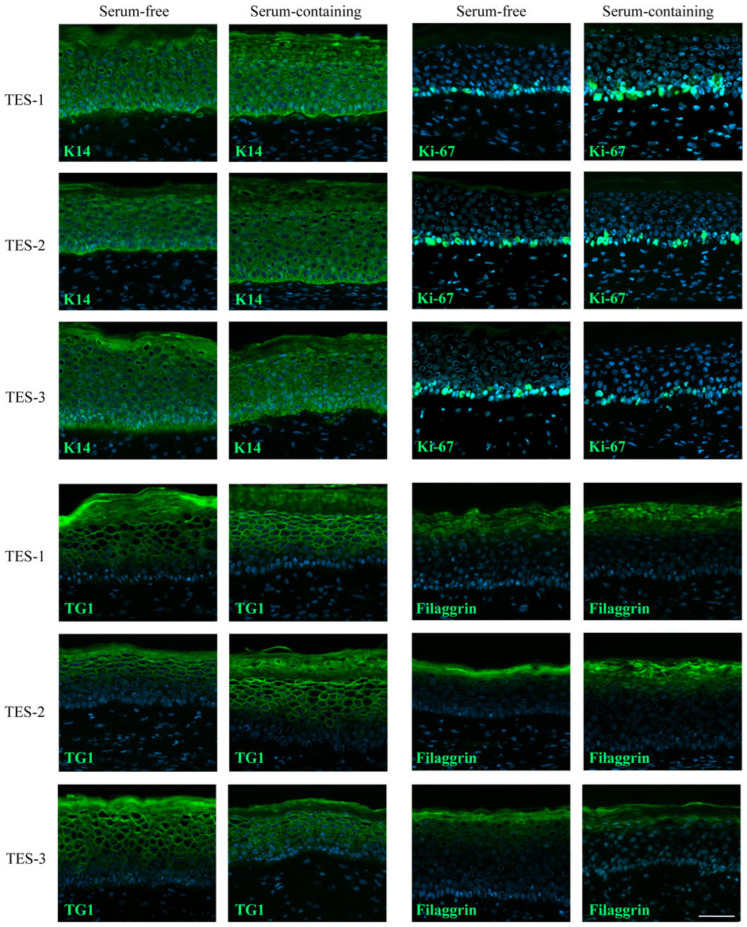
Analysis of skin markers in TES-1, TES-2, and TES-3 produced with keratinocytes and fibroblasts from different donors (see Table 1), matured in vitro in serum-free or serum-containing media. Immunofluorescence staining against keratin 14 (K14), Ki-67, transglutaminase 1 (TG1), and filaggrin. Cell nuclei were stained with Hoechst reagent. The quantification of image signal is presented in Figure 4. Scale bar: 50 μm.

**Figure 4:**
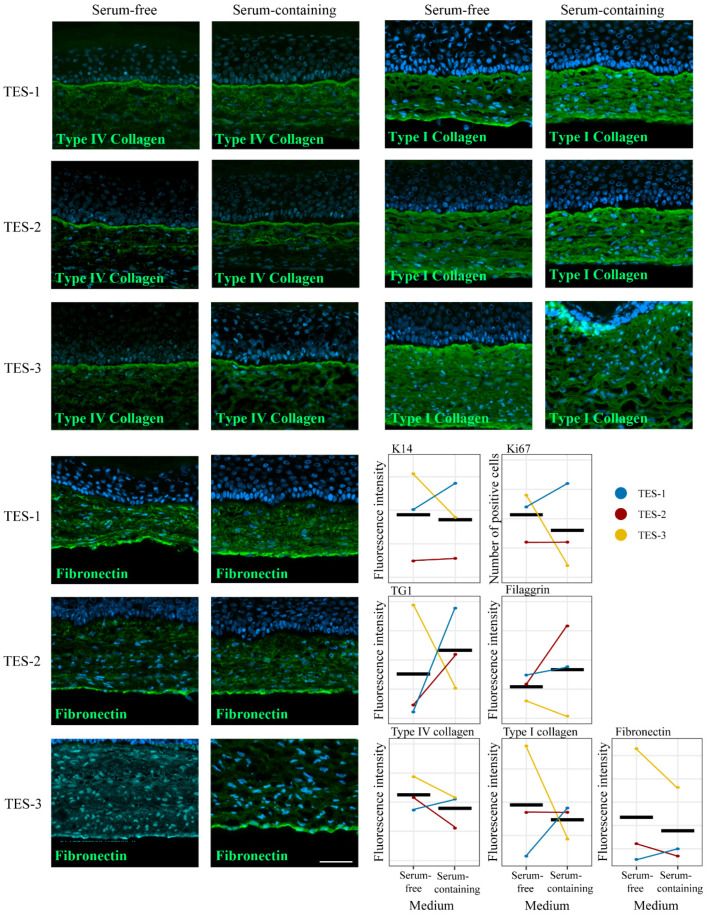
Analysis of skin markers in TES-1, TES-2, and TES-3 produced with keratinocytes and fibroblasts from different donors (see Table 1), matured in vitro in serum-free or serum-containing media. Immunofluorescence staining against type IV collagen, type I collagen, and fibronectin. Cell nuclei were stained with Hoechst reagent. Scale bar: 50 μm. Fluorescence intensity was measured using ImageJ 53e software [25] for markers from Figure 3 and Figure 4. The solid black line represents the mean. Linear mixed-effect model with paired samples was used to compare the effect of media on marker expression. No statistically significant effect of condition was detected (*p* > 0.05).

In order to identify any other potential differences between TESs produced with serum-free and serum-containing media, immunofluorescence stainings were performed against various epidermal and dermal markers. The expression of keratin 14 (K14), a basal epithelial cell marker; Ki-67, a proliferation marker; transglutaminase (TG1), a differentiation marker; filaggrin, a skin differentiation marker; type IV collagen (Col IV), an essential component of the basement membrane; and type I collagen and fibronectin, two components of the dermal extracellular matrix, were assessed by immunofluorescence (Figure 3 and Figure 4). For all these labelings, the signal was quantified (Figure 4), and there was no significant difference in protein expression and localization between TESs produced with either type of media (Figure 3 and Figure 4). These results suggest that TESs produced with serum-free or serum-containing media did not display phenotypic or structural differences and expressed in a similar fashion markers usually found in normal human skin. We show here, unlike other studies, that it is possible to successfully produce TESs that express normal human skin markers using serum-free media [22]. One possible reason for this discrepancy is that, unlike commercially available serum-free media that contain low calcium concentrations, the calcium content of our serum-free medium is sufficient to allow the formation of desmosomes and a cohesive differentiated epidermis. Indeed, keratinocytes cultured in low calcium concentrations grow as a monolayer. Calcium also impacts the proliferation and differentiation of keratinocytes [23], and it is essential for the formation of desmosomes [24].

### 2.3. Contraction Assessment

**Figure 5:**
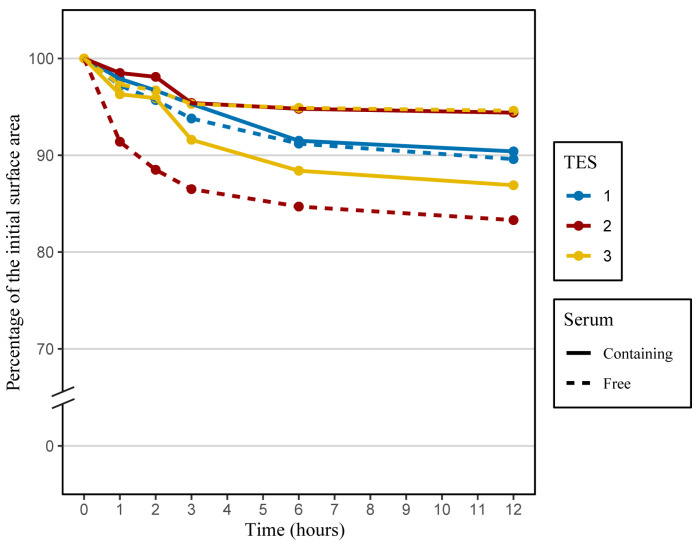
Contractile behavior of TES-1, TES-2, and TES-3 produced with keratinocytes and fibroblasts from different donors (see Table 1), matured in vitro. TESs produced with serum-free medium are in dashed lines, and TESs produced with serum-containing medium are in solid lines. TES-1 (blue curves), TES-2 (red curves), and TES-3 (yellow curves) were placed on an agar substrate, and the surface area of the skin substitutes was measured over time to obtain contraction kinetic curves; n = 3.

Next, we conducted a comparison of the structural stability of TESs produced with serum-containing or serum-free media. Anticipating tissue response to certain physical stimuli is advantageous in a clinical context. Indeed, mechanical resistance of skin substitutes should be sufficient to sustain manipulations by surgeons and to resist subsequent mechanical stress after grafting. Moreover, tissue contraction before or after grafting will affect the final TES surface area available for wound coverage and may impact scar formation. Previous studies have indicated that most TES contractions occur within the first few hours after detachment from their anchorage [2]. Thus, TES contraction was followed over a 12 h period after anchorage detachment at the end of the culture at the air–liquid interface. As expected, most of the contractions took place within the first three hours, and the extent of contraction varied depending on the cell populations and media used to produce TESs. The mean percentage of contraction for TESs produced with serum-free medium was 10.8%, while it was 9.4% for TESs produced with serum-containing medium after a 12 h period (Figure 5). There was no significant difference in the contraction for TESs produced with media containing serum or not. A previous study showed a correlation between the presence of serum in the culture medium and the intensity and rapidity of fibroblast contraction. Indeed, it has been shown that fibroblasts cultured within a fibrin gel will result in a higher contraction of the gel when cultured in the presence of serum. After 4 h, there was a reduction of 70% in gel thickness when fibroblasts were cultured with 10% serum, compared to 50% when fibroblasts were cultured without serum [26]. Another study also showed that proteins contained in serum are necessary for the contraction of collagen lattices by human skin fibroblasts [27]. Our model is different from fibrin or collagen gels, since fibroblasts have produced and organized their own extracellular matrix. Moreover, the presence of an epidermis has a significant impact on TES contraction. A terminally differentiated epidermis reduces by 2.2 fold the fibroblast’s contractile behavior [28].

### 2.4. Mechanical Properties of Skin Substitutes

**Figure 6:**
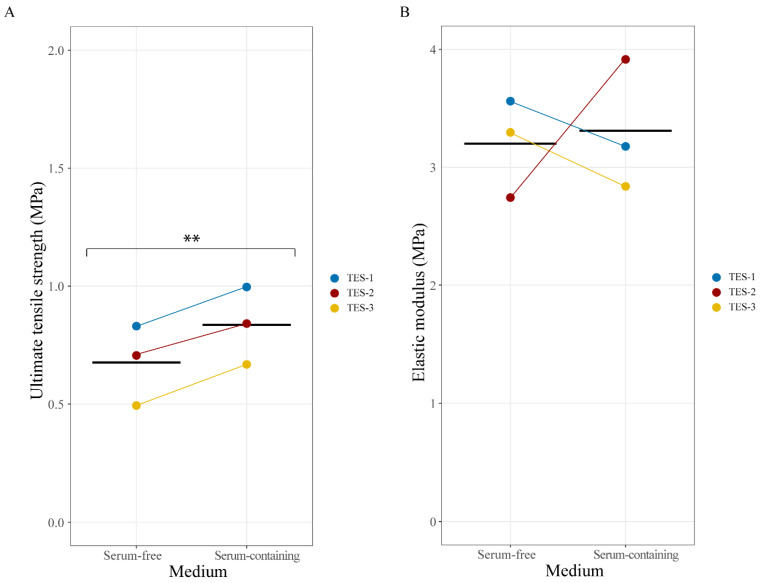
(**A**). Ultimate tensile strength (ETS) and (**B**). elastic modulus (EM) of TES-1, TES-2, and TES-3 produced with keratinocytes and fibroblasts from different donors (see Table 1), matured in vitro in serum-free or serum-containing media. TES-1 is represented in blue; TES-2 is in red; and TES-3 is in yellow; n = 3 replicates for each donor. The solid black line represents the mean. A linear mixed-effect model with paired samples was used to compare the effect of media on mechanical properties. ** *p* < 0.01.

Skin substitutes must possess a minimal amount of stretching resistance to enable easy manipulation by surgeons and to follow the patient’s movements. Therefore, we measured the ultimate tensile strength (UTS) and elastic modulus of the TESs produced with either type of media using a standard tensile test. UTS values were increased by 0.158 units on average in TESs produced in the serum-containing condition when compared to those produced in the serum-free medium (*p* = 0.0056; Figure 6A). Slight variations in the values of the elastic modulus were observed between TESs from different populations and depending on the presence or absence of serum. However, no statistically significant difference was observed (*p* = 0.8086; Figure 6B). Our results are in line with previous studies concerning self-assembled skin substitutes’ mechanical properties [29,30]. Both serum-containing and serum-free TES mechanical properties fall inside the intrinsic variability of the self-assembly method (between 1 and 5 MPa for elastic modulus and between 0.2 and 1 MPa for UTS) as well as values measured for the dermal tissue-engineered substitutes (stromal portion only) produced using serum-free medium [18]. This finding indicates that even if some tendencies may be perceptible, changes in UTS and elastic modulus might not be biologically significant. Since TESs produced with serum-containing media are already used in clinical trials and show sufficient tensile properties and promising results [2], our results show that serum-free TESs have similar, hence sufficient, tensile strength to be grafted onto patients.

### 2.5. In Vivo Assessment

In the next phase of our study, we conducted an in vivo experiment to assess the ability of the epidermis of TESs produced with serum-free media to sustain epithelial turnover for a period of six months. This is a critical aspect, as it indicates the preservation of functional epithelial stem cells over time. Thus, TESs produced with both types of media were grafted onto athymic mice.

**Figure 7:**
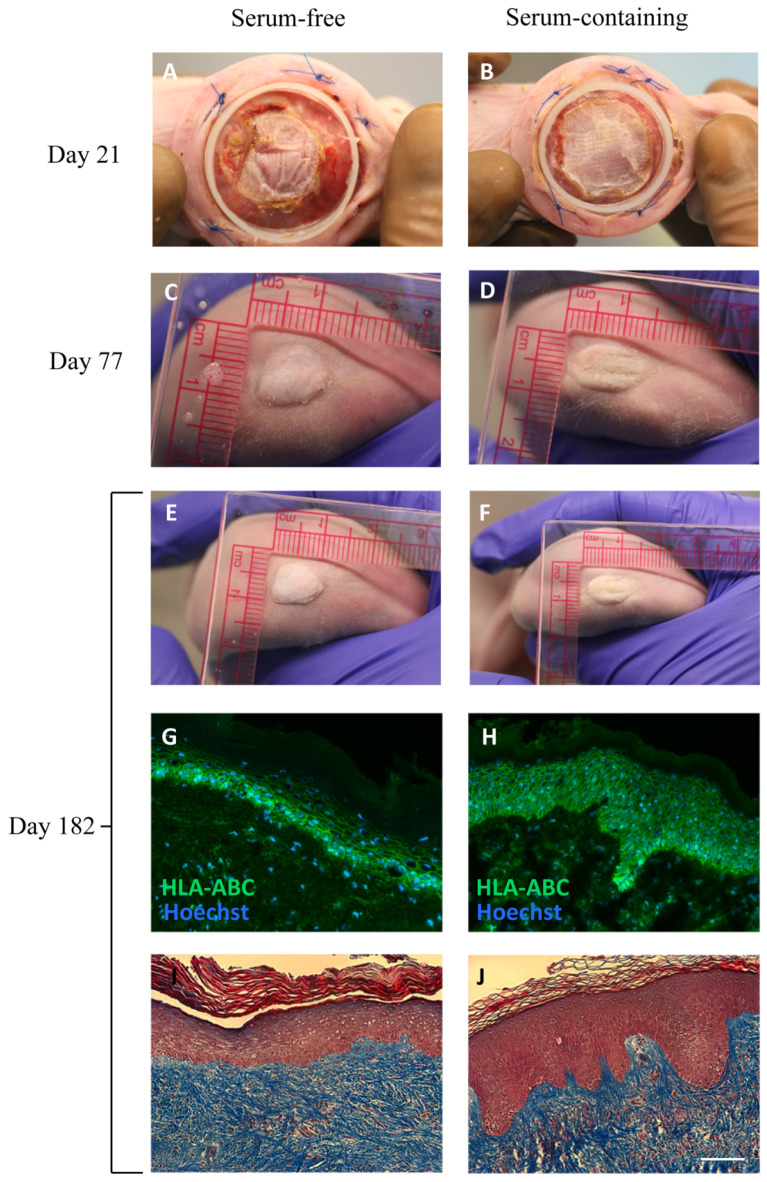
Macroscopic aspect (**A**–**F**), immunolabeling for the human histocompatibility complex HLA-ABC (**G**,**H**), and histological analysis (**I**,**J**) of TES-4 produced with serum-free or serum-containing media and grafted on athymic mice, at 21, 77, and 182 days. Scale bar (**G**–**J**): 100 μm.

TESs produced with both types of media showed a good graft adherence and graft persistence throughout the entire six-month duration of the experiment (Figure 7). There were no significant observable differences between TESs produced with either type of media. At the end of the six-month experiment, TESs appeared uniform and smooth, resembling normal human skin. There was no difference between both types of media; TESs persisted for the entire grafting period in 100% of the mice. The positive human leukocyte antigen (HLA-ABC) labeling of TESs confirmed the human origin of the skin harvested six months after grafting onto mice and suggests that functional epithelial stem cells were preserved within the TESs. An epidermis with normal histology was present on all grafts 182 days after grafting. A number of factors can contribute to stem cell retention in skin epithelial cell cultures, such as the use of a feeder layer or the use of serum [31]. Previous clinical studies suggested that TESs produced with serum or a serum replacement may result in a better persistence after grafting onto humans than TESs produced in serum-free conditions [6]. The results displayed in the present in vivo study indicate that TESs can be successfully achieved under serum-free conditions and can persist long-term after grafting.

## 3. Materials and Methods

### 3.1. Ethical Considerations

These experiments were conducted in agreement with the Declaration of Helsinki and our institution’s guidelines. This study was approved by our institution’s protection of human participants (Comité d’éthique de la recherche du CHU de Québec-Université Laval: no. 2012-1251; 28 April 1992) and animal care (Comité de protection des animaux de l’Université Laval: no. 2018-003; 26 January 2018) committees. Skin donors gave their written informed consent for the use of tissues for research and educational purposes.

### 3.2. Cell Populations

Human cells were obtained from healthy human donors (Table 1). Keratinocytes and dermal fibroblasts were isolated from the excess skin of facelift or breast-reduction surgeries (TES-1, TES-2, and TES-4) of five adult women (18 to 56 years old (two donors for fibroblasts and three donors for keratinocytes)) or from foreskin tissues of a 1-year-old boy (TES-3 (fibroblasts and keratinocytes from the same donor)). Cells were isolated, expanded, frozen after primary culture (p0), and stored in liquid nitrogen in bovine serum containing 10% DMSO, as previously described [21].

### 3.3. Cell Culture

Dermal fibroblasts were thawed and cultured either in Prime XV (FUJIFILM Irvine Scientific, Santa Ana, CA, USA), a commercially available serum-free medium for stromal cells, or in fibroblast culture medium (fDME), which consisted of Dulbecco–Vogt modified Eagle medium (Thermo Fisher Scientific, Ottawa, ON, Canada) supplemented with 10% fetal bovine serum (FBS; Seradigm), 100 U/mL penicillin (Pharmaceutical Partners of Canada, Inc., Richmond Hill, ON, Canada), and 25 μg/mL gentamycin (Galenova, St-Hyacinthe, QC, Canada). Plates used for fibroblasts cultured in serum-free medium (Prime XV) were coated with human fibronectin to promote cell adhesion on the plastic substrate [18].

Keratinocytes were thawed and grown on a feeder layer of irradiated human dermal fibroblasts. Plates used for keratinocytes cultured in serum-free medium (Surge SFM) were coated with human fibronectin [17]. Keratinocytes were cultured in Surge SFM or in keratinocyte culture medium (ckDME-Ham), which consisted of Dulbecco–Vogt modified Eagle medium: Ham’s F12, ratio 3:1, 24.3 μg/mL adenine (Corning, Glendale, CA, USA), supplemented with 5 μg/mL insulin (Sigma-Aldrich, Saint-Louis, MO, USA), 1.1 mM hydrocortisone (Novapharm), 0.212 μg/mL isoproterenol hydrochloride (Sandoz, Boucherville, QC, Canada), 5% bovine Fetal Clone II serum (HyClone, South Logan, UT, USA), 10 ng/mL human epidermal growth factor (EGF, R&D Systems, Minneapolis, MN, USA), 100 U/mL penicillin, and 25 μg/mL gentamycin, as previously described [21]. When keratinocytes reached 80–95% confluence, they were trypsinized and seeded on tissue-engineered fibroblast sheets. Cell counts and cell size measurements were performed with a Coulter automated cell counter (Beckman Coulter, Brea, CA, USA).

### 3.4. Skin Substitute Production

To produce TESs, fibroblasts were subcultured for one passage after thawing. They were then trypsinized and seeded in 85 cm^2^ Nunc^TM^ Omnitray^TM^ tissue culture plates at a density of 4 × 10^3^ cells/cm^2^ in fibroblast medium. Ascorbic acid (Sigma-Aldrich, Saint-Louis, MO, USA) was added at a concentration of 50 μg/mL in the serum-containing medium to promote extracellular matrix production. It was not necessary to add ascorbic acid to the Prime XV medium [18]. Medium was changed three times per week for 21 days until tissue-engineered fibroblast sheets were formed.

Keratinocytes were seeded at passage 2 directly on top of a tissue-engineered fibroblast sheet at a density of 1 × 10^5^ cells/cm^2^ in keratinocyte medium. The culture medium was supplemented with 50 μg/mL ascorbic acid and changed every day for four days. The day after keratinocyte seeding, custom-made frames, cut out of Ahlstrom grade 237 filter paper (Fisher Scientific, Hampton, VA, USA), were placed onto 21-day tissue-engineered fibroblast sheet containing keratinocytes. Small sterile metal ingots were added onto the frames to hold them in place. After three days, medium was removed, and the tissue surrounding the frame was folded over the frame using sterile dissection forceps. Each framed tissue sheet was slowly lifted from the culture plate and stacked onto a subsequent tissue-engineered fibroblast sheet. Surrounding tissue was once again folded over the paper frame, and the same process was repeated one more time. The three-sheet constructs were then attached to the frame with LIGACLIPS^®^ (Ethicon Endo-Surgery, Raritan, NJ, USA). The substitutes were then placed on polypropylene membranes (Spectrum Labs, New Brunswick, NJ, USA) laid on a custom-made acrylic support to hold them at the air–liquid interface. The substitutes were cultured for 10 additional days in the same medium without EGF.

In total, 36 TESs were produced: 18 with serum-free media and 18 with serum-containing media. Two pieces (25 mm × 25 mm)/TES were cut from twelve TESs (6 with serum and 6 with serum-free media) and grafted onto nude mice (see Section 3.8). As for the other 24 TESs, a 30 mm × 30 mm piece was cut for the contraction test; a 36 mm × 11 mm piece was used for the traction test; and the rest were used for histological and immunofluorescence analyses.

### 3.5. Histological and Immunofluorescence Analyses

TES biopsies harvested prior to and after grafting were fixed overnight in formol 3.7% and embedded in paraffin. Sections were then stained with Masson’s trichrome or with hematoxylin and eosin as described in [21].

Biopsies of TES were also embedded in Tissue-Tek optimal cutting temperature Compound (Sakura Finetek, Torrance, CA, USA), frozen in liquid nitrogen, and stored at −80 °C. Immunofluorescence assays were carried out on 5 µm thick cryosections fixed with acetone (10 min at −20 °C). Sections were washed with phosphate-buffered saline (PBS) and then incubated with a blocking buffer (1% *w*/*v* bovine serum albumin (BSA) in PBS) for 30 min. Following PBS rinses, tissue sections were incubated for 45 min with primary antibodies diluted in 1% *w*/*v* BSA in PBS. The following primary antibodies were used: rabbit anti-keratin 14 1:800 (K14; Cedarlane, Burlington, ON, Canada), anti-type IV collagen 1:400 (COL IV; Abcam, Cambridge, UK), and anti-transglutaminase 1 (TG1) 1:200 (Protein Tech, Rosemont, IL, USA) antibodies; mouse monoclonal anti-Ki-67 1:200 (BD Biosciences, San Jose, CA, USA), anti-filaggrin 1:800 (Santa Cruz, Dallas, TX, USA), anti-fibronectin 1:600 (Abcam, Cambridge, UK), anti-type I collagen 1:80 (Cedarlane, Burlington, ON, Canada), and anti-human leukocyte antigen A, B, C 1:25 (HLA-ABC; Biolegend, San Diego, CA, USA) antibodies. Following PBS rinses, tissues were incubated for 30 min with the following secondary antibodies: Alexa 488-conjugated goat anti-mouse antibody 1:800 (Invitrogen, Waltham, MA, USA) or donkey anti-rabbit 1:800 (Invitrogen, Waltham, MA, USA). Cell nuclei were stained with Hoechst (Sigma-Aldrich, Saint-Louis, MO, USA). The fluorescence intensities of K14, TG1, filaggrin, type IV collagen, type I collagen, and fibronectin were measured using ImageJ 1.53e software [25]. For Ki-67, the number of positive cells in the basement membrane was counted for each condition. Three pictures (magnification 20×) were analyzed per condition.

### 3.6. Contraction Tests

The structural stability of the skin substitutes was assessed at the end of the culture period (after 10 days of culture at the air–liquid interface) by measuring the tissue contraction of TESs lying on an agar gel, as previously described [28]. Briefly, a sterile non-adherent gauze was used to transfer the TES onto a sterile cutting board. Biopsies were cut out under sterile conditions using an in-house-designed stainless-steel die (30 × 30 mm^2^). The biopsies were then transferred onto a gel composed of 1% *w*/*v* agar in DME. To evaluate the contractile behavior of TESs, pictures were taken at 0, 1, 2, 3, 6, and 12 h. These pictures were analyzed using ImageJ 1.53e software [25] to evaluate the variation of the tissue surface area over time. All measurements were expressed as a ratio of the initial surface area of the TES biopsy. Measurements were taken in triplicate (from three different TESs) for each population and each medium.

### 3.7. Tensile Strength Tests

Mechanical properties of the skin substitutes were assessed at the end of the culture period using a universal tensile testing machine (Instron Corp., Norwood, MA, USA). A custom-made punch was used to cut out a dog-bone-shaped specimen of the sample. The specimen was then mounted on the machine and stretched at a constant rate of 0.2 mm/s until failure, while the force measured by the load cell was recorded as a function of displacement. The thickness of each sample was measured on histological slides using ImageJ 1.53e software [25], and the width of the punch was considered as the width of the specimen. Force-displacement data were transformed into a stress–strain curve using Matlab and dimensions of the specimen. The highest stress value and the slope of the linear part of the curve corresponded to the ultimate tensile strength (UTS) and the elastic modulus, respectively. There were three biological replicates per type of medium, and measurements were taken on three samples for each of them.

### 3.8. Skin Substitute Grafting on Nude Mice

TES-4 samples were grafted onto the backs of CD1-Foxn1 nude mice (Charles River Laboratories, Wilmington, NC, USA), as previously described [32,33]. Briefly, a portion of the mid-back skin of the mouse was removed. Then, the fascicular panniculus was removed with forceps to expose muscle, and a custom-made silicone Fusenig’s chamber was inserted around the wound to prevent contraction of the mouse skin. The Fusenig’s chamber was secured in place by four intramuscular sutures (Prolene 4-0, Ethicon, Raritan, NJ, USA). A 5 cm^2^ biopsy was taken from the skin substitutes cultured for 10 days at the air–liquid interface, mounted on a non-adhering dressing (AdapticTM, Acelity, San Antonio, TX, USA), and grafted into the aperture. To prevent graft displacement, sterile gauzes were placed over the substitute inside the Fusenig’s chamber and served as a bolus tie-over pressure dressing. The gauzes were taken out after 7 days. The silicone chambers were removed 21 days after grafting. Three to four mice per condition at each time point were euthanized 3 weeks, 3 months, and 6 months after grafting for tissue collection and analysis.

### 3.9. Statistical Analyses

Statistical analyses were carried out using R (v3.6.1, R Studio v1.1.456). Linear mixed-effect models with paired samples were fitted using the nlme package [34]. Homoskedasticity and normality of residuals were verified with residuals over the fitted value and QQ plots, respectively. The significance threshold was set at 0.05.

## 4. Conclusions

The findings from this study provide compelling evidence that TESs can be successfully produced using serum-free media, and they closely resemble TESs produced with conventional serum-containing media. TESs produced with both types of media demonstrated similar macroscopic appearance, phenotypic characteristics, histological structure, protein expression, and mechanical properties. We also showed that long-term grafting of TESs produced with serum-free media on nude mice yields similar results to TESs produced with serum-containing media over a six-month period.

The replacement of serum-containing media with serum-free media for the production of TESs in a clinical setting could represent a significant advancement in terms of biosafety and reproducibility if its security is confirmed through further testing, such as karyotype stability assessment. In conclusion, the use of Prime XV and Surge SFM holds promise for producing skin substitutes for basic research applications and to produce autologous TESs that could be tested further for clinical research. The next steps involve testing the entire protocol using serum-free media, from cell extraction to TESs production.

## Figures and Tables

**Table 1:** List of the cell populations used in this study.

TES	Keratinocytes	Donor Age (Years)	Fibroblasts	Donor Age (Years)
TES-1	K1	D1 (37)	F1	D5 (18)
TES-2	K2	D2 (26)	F2	D6 (18)
TES-3	K3	D3 (1)	F3	D3 (1)
TES-4	K4	D4 (56)	F4	D6 (18)

## Data Availability

The data presented in this study are available on request from the corresponding author.
